# Supplementary material for: Carvacrol Microemulsion vs. Nanoemulsion as Novel Pork Minced Meat Active Coatings
Source: Nanomaterials (Basel). 2023 Dec 18;13(24):3161. doi: 10.3390/nano13243161 (PMC10745327; doi:10.3390/nano13243161)
Supplement: Supplementary file 1 [file nanomaterials-13-03161-s001.zip › nanomaterials-2745879-supplementary.pdf]

Supplementary Materials

Carvacrol Microemulsion vs. Nanoemulsion as Novel Pork Minced Meat Active Coatings

Konstantinos Zaharioudakis [1], Eleni Kollia [2], Areti Leontiou [1], Dimitrios Moschovas [3], Andreas Karydis-Messinis [3], Apostolos Avgeropoulos [3], Nikolaos E. Zafeiropoulos [3], Efthymia Ragkava [1], George Kehayias [1],
Charalampos Proestos [2], Constantinos E. Salmas [3,*] and Aris E. Giannakas [1,*]

[1] Department of Food Science and Technology, University of Patras, 30100 Agrinio, Greece; zacharioudakis.k@upatras.gr (K.Z.); aleontiu@upatras.gr (A.L.); effierag@yahoo.com (E.R.); gkechagi@upatras.gr (G.K.)
[2] Laboratory of Food Chemistry, Department of Chemistry, National and Kapodistrian University of Athens Zografou, 15771 Athens, Greece; elenikollia@chem.uoa.gr (E.K.); harpro@chem.uoa.gr (C.P.)
[3] Department of Material Science and Engineering, University of Ioannina, 45110 Ioannina, Greece;dmoschov@uoi.gr (D.M.); a.karyd@uoi.gr (A.K.-M.); aavger@uoi.gr (A.A.); nzafirop@uoi.gr (N.E.Z.)
* Correspondence: ksalmas@uoi.gr (C.E.S.); agiannakas@upatras.gr (A.E.G.)

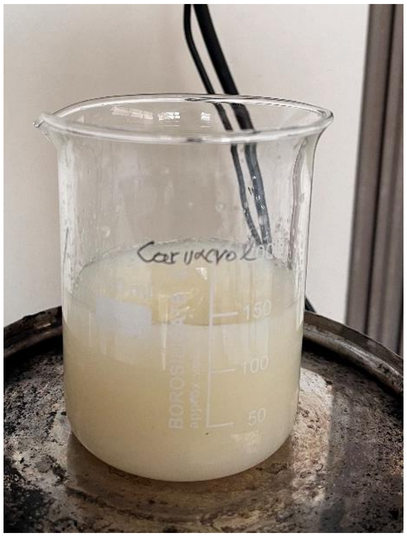

Figure S1. Preparation of carvacrol nanoemulsion (NC)

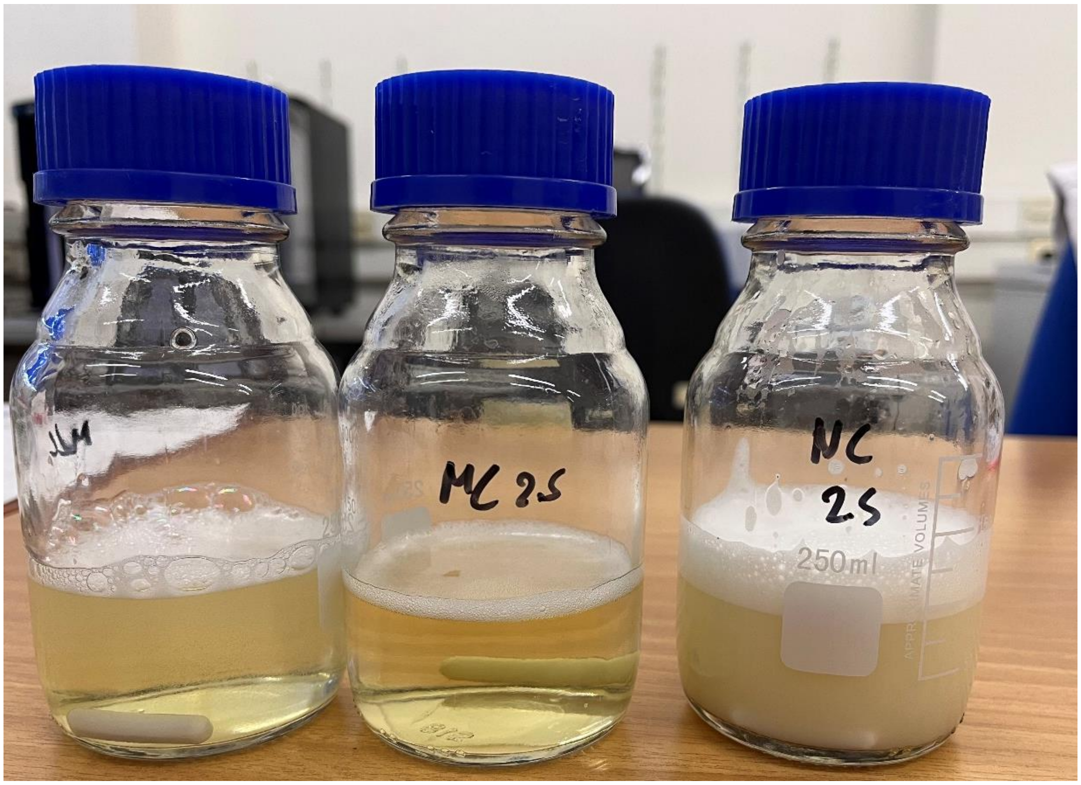

Figure S2. Image with Carvacrol microemulsion (MC)(left), carvacrol microemulsion with chitosan (MC)(middle) and carvacrol nanoemulsion (NC)(right) prepared in this study.

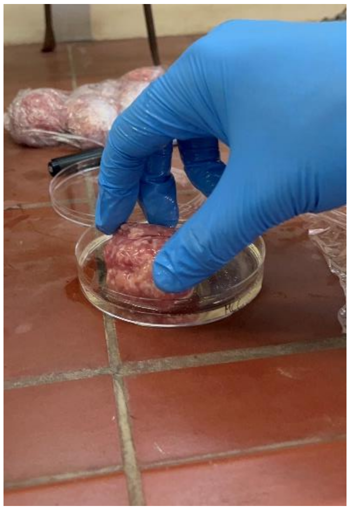

Figure S3. Minced meat coating process

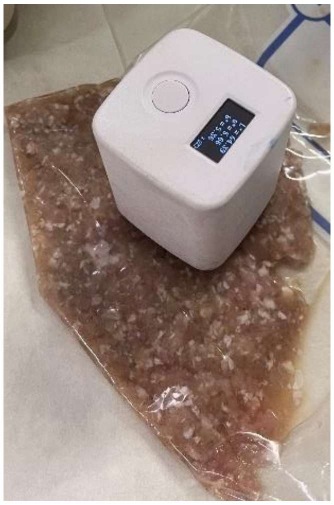

Figure S4. Representative image with L*a*b measurement in minced pork meat samples

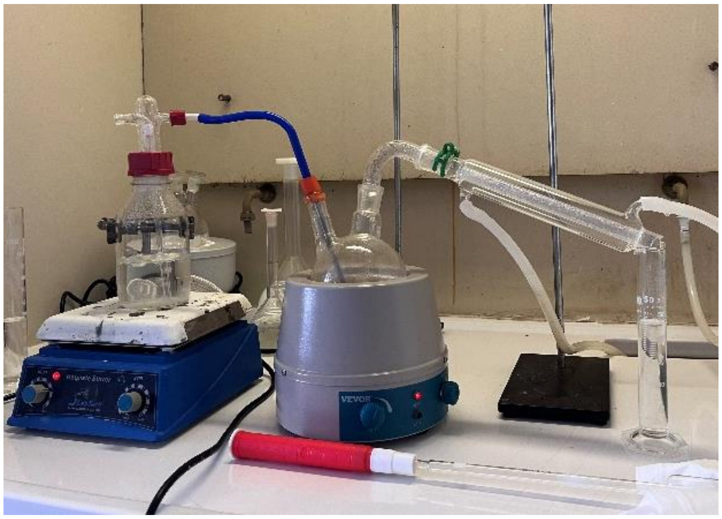

Figure S5. Steam distillation apparatus employed for the calculation of TBARs in meat samples.

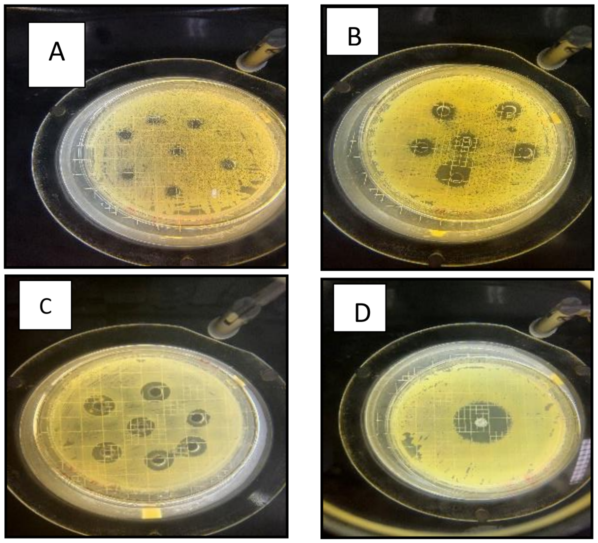

Figure S6. Representative images of obtained diffusion zone: A) FC, **B**) MC, C) MCC and D) NC samples

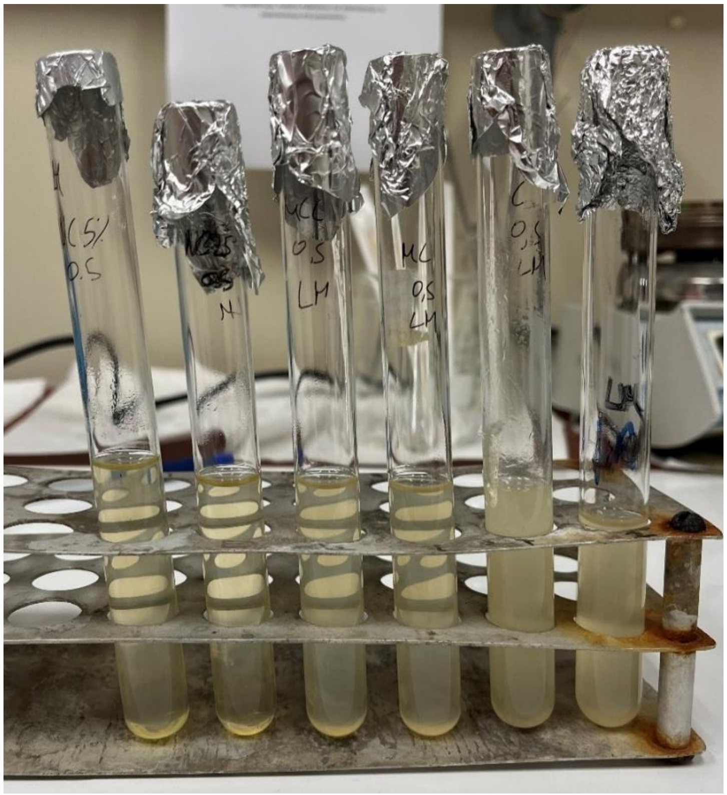

Figure S7. Representative image with the result of MIC in Carvacrol Treatments against *Listeria monocytogenes*

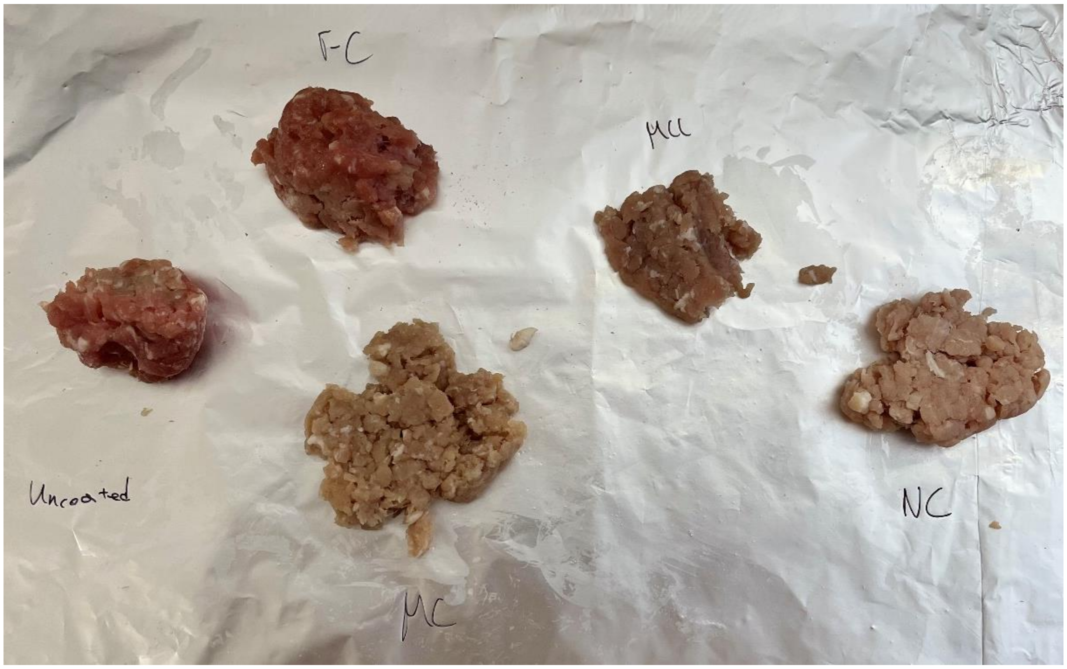

Figure S8. Minced meat samples after 9 days of storage at 4 °C.

Table S1 Non-parametric in Inhibition Zones

Hypothesis Test Summary

	Null Hypothesis	Test	Sig.[a,b]	Decision
1	The distribution of Listeria is the same across categories of Treatment.	Independent-Samples Kruskal-Wallis Test	0.016	Reject the null hypothesis.
2	The distribution of Saureus is the same across categories of Treatment.	Independent-Samples Kruskal-Wallis Test	0.016	Reject the null hypothesis.

a. The significance level is ,050.

b. Asymptotic significance is displayed.

Pairwise Comparisons of Treatment

Sample 1-Sample 2	Test Statistic	Std. Error	Std. Test Statistic	Sig.	Adj. Sig.[a]
FC-MC	-3.000	2.944	-1.019	0.308	1.000
FC-MCC	-6.000	2.944	-2.038	0.042	0.249
FC-NC	-9.000	2.944	-3.057	0.002	0.013
MC-MCC	-3.000	2.944	-1.019	0.308	1.000
MC-NC	-6.000	2.944	-2.038	0.042	0.249
MCC-NC	-3.000	2.944	-1.019	0.308	1.000

Each row tests the null hypothesis that the Sample 1 and Sample 2 distributions are the same.
Asymptotic significances (2-sided tests) are displayed. The significance level is ,050.
a. Significance values have been adjusted by the Bonferroni correction for multiple tests.

Table S2 pH statistical analysis

Day_0 across Treatment

Independent-Samples Kruskal-Wallis Test Summary

Total N	15
Test Statistic	7,943[a]
Degree Of Freedom	4
Asymptotic Sig.(2-sided test)	0.094

a. The test statistic is adjusted for ties.

Pairwise Comparisons of Treatment

Sample 1-Sample 2	Test Statistic	Std. Error	Std. Test Statistic	Sig.	Adj. Sig.[a]
MCC-FC	6.167	3.632	1.698	0.090	0.895
MCC-NC	-6.333	3.632	-1.744	0.081	0.812
MCC-MC	8.000	3.632	2.203	0.028	0.276
MCC-Uncoated	9.500	3.632	2.616	0.009	0.089
FC-NC	-0.167	3.632	-0.046	0.963	1.000
FC-MC	-1.833	3.632	-0.505	0.614	1.000
FC-Uncoated	3.333	3.632	0.918	0.359	1.000
NC-MC	1.667	3.632	0.459	0.646	1.000
NC-Uncoated	3.167	3.632	0.872	0.383	1.000
MC-Uncoated	1.500	3.632	0.413	0.680	1.000

Each row tests the null hypothesis that the Sample 1 and Sample 2 distributions are the same.
Asymptotic significances (2-sided tests) are displayed. The significance level is ,050.
a. Significance values have been adjusted by the Bonferroni correction for multiple tests.

Day_3 across Treatment

Independent-Samples Kruskal-Wallis Test Summary

Total N	15
Test Statistic	12,633[a]

Degree Of Freedom	4
Asymptotic Sig.(2-sided test)	0.013

a. The test statistic is adjusted for ties.

Pairwise Comparisons of Treatment

Sample 1-Sample 2	Test Statistic	Std. Error	Std. Test Statistic	Sig.	Adj. Sig.[a]
MCC-NC	-3.333	3.651	-0.913	0.361	1.000
MCC-FC	5.667	3.651	1.552	0.121	1.000
MCC-Uncoated	10.000	3.651	2.739	0.006	0.062
MCC-MC	11.000	3.651	3.012	0.003	0.026
NC-FC	2.333	3.651	0.639	0.523	1.000
NC-Uncoated	6.667	3.651	1.826	0.068	0.679
NC-MC	7.667	3.651	2.100	0.036	0.358
FC-Uncoated	4.333	3.651	1.187	0.235	1.000
FC-MC	-5.333	3.651	-1.461	0.144	1.000
Uncoated-MC	-1.000	3.651	-0.274	0.784	1.000

Each row tests the null hypothesis that the Sample 1 and Sample 2 distributions are the same.
Asymptotic significances (2-sided tests) are displayed. The significance level is ,050.
a. Significance values have been adjusted by the Bonferroni correction for multiple tests.

Day_6 across Treatment

Independent-Samples Kruskal-Wallis Test Summary

Total N	15
Test Statistic	12,982[a]
Degree Of Freedom	4
Asymptotic Sig.(2-sided test)	0.011

a. The test statistic is adjusted for ties.

Pairwise Comparisons of Treatment

Sample 1-Sample 2	Test Statistic	Std. Error	Std. Test Statistic	Sig.	Adj. Sig.[a]
MCC-NC	-1.333	3.648	-0.365	0.715	1.000
MCC-FC	5.167	3.648	1.416	0.157	1.000
MCC-Uncoated	8.167	3.648	2.239	0.025	0.252
MCC-MC	11.167	3.648	3.061	0.002	0.022
NC-FC	3.833	3.648	1.051	0.293	1.000
NC-Uncoated	6.833	3.648	1.873	0.061	0.611
NC-MC	9.833	3.648	2.695	0.007	0.070
FC-Uncoated	3.000	3.648	0.822	0.411	1.000
FC-MC	-6.000	3.648	-1.645	0.100	1.000
Uncoated-MC	-3.000	3.648	-0.822	0.411	1.000

Each row tests the null hypothesis that the Sample 1 and Sample 2 distributions are the same.
Asymptotic significances (2-sided tests) are displayed. The significance level is ,050.
a. Significance values have been adjusted by the Bonferroni correction for multiple tests.

Day_9 across Treatment

Independent-Samples Kruskal-Wallis Test Summary

Total N	15

Test Statistic	13,500[a]
Degree Of Freedom	4
Asymptotic Sig.(2-sided test)	0.009

a. The test statistic is adjusted for ties.

Pairwise Comparisons of Treatment

Sample 1-Sample 2	Test Statistic	Std. Error	Std. Test Statistic	Sig.	Adj. Sig.[a]
NC-FC	3.000	3.651	0.822	0.411	1.000
NC-MCC	6.000	3.651	1.643	0.100	1.000
NC-MC	9.000	3.651	2.465	0.014	0.137
NC-Uncoated	12.000	3.651	3.286	0.001	0.010
FC-MCC	-3.000	3.651	-0.822	0.411	1.000
FC-MC	-6.000	3.651	-1.643	0.100	1.000
FC-Uncoated	9.000	3.651	2.465	0.014	0.137
MCC-MC	3.000	3.651	0.822	0.411	1.000
MCC-Uncoated	6.000	3.651	1.643	0.100	1.000
MC-Uncoated	3.000	3.651	0.822	0.411	1.000

Each row tests the null hypothesis that the Sample 1 and Sample 2 distributions are the same.

Asymptotic significances (2-sided tests) are displayed. The significance level is ,050.

a. Significance values have been adjusted by the Bonferroni correction for multiple tests.

Table S3 Non-parametric in Lipid Oxidation Analysis in pork minced meat for 9 days

Hypothesis Test Summary

	Null Hypothesis	Test	Sig.[a,b]	Decision
1	The distribution of Day_0 is the same across categories of Treatment.	Independent-Samples Kruskal-Wallis Test	0.374	Retain the null hypothesis.
2	The distribution of Day_3 is the same across categories of Treatment.	Independent-Samples Kruskal-Wallis Test	0.032	Reject the null hypothesis.
3	The distribution of Day_6 is the same across categories of Treatment.	Independent-Samples Kruskal-Wallis Test	0.018	Reject the null hypothesis.
4	The distribution of Day_9 is the same across categories of Treatment.	Independent-Samples Kruskal-Wallis Test	0.032	Reject the null hypothesis.

a. The significance level is ,050.

b. Asymptotic significance is displayed.

Day 0 across treatment

Independent-Samples Kruskal-Wallis Test Summary

Total N	15
Test Statistic	4,242[a]
Degree Of Freedom	4
Asymptotic Sig.(2-sided test)	0.374

a. The test statistic is adjusted for ties.

Pairwise Comparisons of Treatment

Sample 1-Sample 2	Test Statistic	Std. Error	Std. Test Statistic	Sig.	Adj. Sig.[a]

MCC-NC	-0.333	3.619	-0.092	0.927	1.000
MCC-Uncoated	3.000	3.619	0.829	0.407	1.000
MCC-MC	5.167	3.619	1.428	0.153	1.000
MCC-FC	5.667	3.619	1.566	0.117	1.000
NC-Uncoated	2.667	3.619	0.737	0.461	1.000
NC-MC	4.833	3.619	1.336	0.182	1.000
NC-FC	5.333	3.619	1.474	0.141	1.000
Uncoated-MC	-2.167	3.619	-0.599	0.549	1.000
Uncoated-FC	-2.667	3.619	-0.737	0.461	1.000
MC-FC	0.500	3.619	0.138	0.890	1.000

Each row tests the null hypothesis that the Sample 1 and Sample 2 distributions are the same.
Asymptotic significances (2-sided tests) are displayed. The significance level is ,050.
a. Significance values have been adjusted by the Bonferroni correction for multiple tests.

Day3

Independent-Samples Kruskal-Wallis Test Summary

Total N	15
Test Statistic	10,561[a]
Degree Of Freedom	4
Asymptotic Sig.(2-sided test)	0.032

a. The test statistic is adjusted for ties.

Pairwise Comparisons of Treatment

Sample 1-Sample 2	Test Statistic	Std. Error	Std. Test Statistic	Sig.	Adj. Sig.[a]
NC-MCC	0.833	3.622	0.230	0.818	1.000
NC-MC	3.500	3.622	0.966	0.334	1.000
NC-Uncoated	8.667	3.622	2.393	0.017	0.167
NC-FC	8.667	3.622	2.393	0.017	0.167
MCC-MC	2.667	3.622	0.736	0.462	1.000
MCC-Uncoated	7.833	3.622	2.163	0.031	0.306
MCC-FC	7.833	3.622	2.163	0.031	0.306
MC-Uncoated	5.167	3.622	1.426	0.154	1.000
MC-FC	5.167	3.622	1.426	0.154	1.000
Uncoated-FC	0.000	3.622	0.000	1.000	1.000

Each row tests the null hypothesis that the Sample 1 and Sample 2 distributions are the same.
Asymptotic significances (2-sided tests) are displayed. The significance level is ,050.
a. Significance values have been adjusted by the Bonferroni correction for multiple tests.

Day6

Independent-Samples Kruskal-Wallis Test Summary

Total N	15
Test Statistic	11,876[a]
Degree Of Freedom	4
Asymptotic Sig.(2-sided test)	0.018

a. The test statistic is adjusted for ties.

Pairwise Comparisons of Treatment

Sample 1-Sample 2	Test Statistic	Std. Error	Std. Test Statistic	Sig.	Adj. Sig.[a]
NC-MCC	3.667	3.645	1.006	0.314	1.000
NC-FC	6.000	3.645	1.646	0.100	0.997
NC-MC	9.000	3.645	2.469	0.014	0.135
NC-Uncoated	11.333	3.645	3.109	0.002	0.019
MCC-FC	2.333	3.645	0.640	0.522	1.000
MCC-MC	5.333	3.645	1.463	0.143	1.000
MCC-Uncoated	7.667	3.645	2.103	0.035	0.354
FC-MC	-3.000	3.645	-0.823	0.410	1.000
FC-Uncoated	5.333	3.645	1.463	0.143	1.000
MC-Uncoated	2.333	3.645	0.640	0.522	1.000

Each row tests the null hypothesis that the Sample 1 and Sample 2 distributions are the same.
Asymptotic significances (2-sided tests) are displayed. The significance level is ,050.
a. Significance values have been adjusted by the Bonferroni correction for multiple tests.

Day_9 across Treatment

Independent-Samples Kruskal-Wallis Test Summary

Total N	15
Test Statistic	10,527[a]
Degree Of Freedom	4
Asymptotic Sig.(2-sided test)	0.032

a. The test statistic is adjusted for ties.

Pairwise Comparisons of Treatment

Sample 1-Sample 2	Test Statistic	Std. Error	Std. Test Statistic	Sig.	Adj. Sig.[a]
NC-MCC	4.167	3.648	1.142	0.253	1.000
NC-MC	6.500	3.648	1.782	0.075	0.748
NC-FC	8.333	3.648	2.284	0.022	0.224
NC-Uncoated	11.000	3.648	3.015	0.003	0.026
MCC-MC	2.333	3.648	0.640	0.522	1.000

MCC-FC	4.167	3.648	1.142	0.253	1.000
MCC-Uncoated	6.833	3.648	1.873	0.061	0.611
MC-FC	1.833	3.648	0.503	0.615	1.000
MC-Uncoated	4.500	3.648	1.233	0.217	1.000
FC-Uncoated	2.667	3.648	0.731	0.465	1.000

Each row tests the null hypothesis that the Sample 1 and Sample 2 distributions are the same.
Asymptotic significances (2-sided tests) are displayed. The significance level is ,050.
a. Significance values have been adjusted by the Bonferroni correction for multiple tests.

Table S4 Non-parametric HSD L value during 9 days of meat storage*

L*

Day0

Independent-Samples Kruskal-Wallis Test Summary

Total N	15
Test Statistic	13,057[a]
Degree Of Freedom	4
Asymptotic Sig.(2-sided test)	0.011

a. The test statistic is adjusted for ties.

Pairwise Comparisons of Treatment

Sample 1-Sample 2	Test Statistic	Std. Error	Std. Test Statistic	Sig.	Adj. Sig.[a]
MC-MCC	-3.000	3.648	-0.822	0.411	1.000
MC-FC	6.667	3.648	1.827	0.068	0.676
MC-Uncoated	8.333	3.648	2.284	0.022	0.224
MC-NC	-12.000	3.648	-3.289	0.001	0.010
MCC-FC	3.667	3.648	1.005	0.315	1.000
MCC-Uncoated	5.333	3.648	1.462	0.144	1.000
MCC-NC	-9.000	3.648	-2.467	0.014	0.136
FC-Uncoated	1.667	3.648	0.457	0.648	1.000
FC-NC	-5.333	3.648	-1.462	0.144	1.000

Uncoated-NC	-3.667	3.648	-1.005	0.315	1.000

Each row tests the null hypothesis that the Sample 1 and Sample 2 distributions are the same.
Asymptotic significances (2-sided tests) are displayed. The significance level is ,050.
a. Significance values have been adjusted by the Bonferroni correction for multiple tests.

Day 3

Independent-Samples Kruskal-Wallis Test Summary

Total N	15
Test Statistic	13,500[a]
Degree Of Freedom	4
Asymptotic Sig.(2-sided test)	0.009

a. The test statistic is adjusted for ties.

Pairwise Comparisons of Treatment

Sample 1-Sample 2	Test Statistic	Std. Error	Std. Test Statistic	Sig.	Adj. Sig.[a]
MCC-MC	3.000	3.651	0.822	0.411	1.000
MCC-FC	6.000	3.651	1.643	0.100	1.000
MCC-Uncoated	9.000	3.651	2.465	0.014	0.137
MCC-NC	-12.000	3.651	-3.286	0.001	0.010
MC-FC	3.000	3.651	0.822	0.411	1.000
MC-Uncoated	6.000	3.651	1.643	0.100	1.000
MC-NC	-9.000	3.651	-2.465	0.014	0.137
FC-Uncoated	3.000	3.651	0.822	0.411	1.000
FC-NC	-6.000	3.651	-1.643	0.100	1.000
Uncoated-NC	-3.000	3.651	-0.822	0.411	1.000

Each row tests the null hypothesis that the Sample 1 and Sample 2 distributions are the same.
Asymptotic significances (2-sided tests) are displayed. The significance level is ,050.
a. Significance values have been adjusted by the Bonferroni correction for multiple tests.

Day6

Independent-Samples Kruskal-Wallis Test Summary

Total N	15
Test Statistic	12,900[a]
Degree Of Freedom	4
Asymptotic Sig.(2-sided test)	0.012

a. The test statistic is adjusted for ties.

Pairwise Comparisons of Treatment

Sample 1-Sample 2	Test Statistic	Std. Error	Std. Test Statistic	Sig.	Adj. Sig.[a]
MCC-FC	4.000	3.651	1.095	0.273	1.000
MCC-Uncoated	5.000	3.651	1.369	0.171	1.000
MCC-MC	9.000	3.651	2.465	0.014	0.137
MCC-NC	-12.000	3.651	-3.286	0.001	0.010
FC-Uncoated	1.000	3.651	0.274	0.784	1.000
FC-MC	-5.000	3.651	-1.369	0.171	1.000
FC-NC	-8.000	3.651	-2.191	0.028	0.285
Uncoated-MC	-4.000	3.651	-1.095	0.273	1.000
Uncoated-NC	-7.000	3.651	-1.917	0.055	0.552
MC-NC	-3.000	3.651	-0.822	0.411	1.000

Each row tests the null hypothesis that the Sample 1 and Sample 2 distributions are the same.
Asymptotic significances (2-sided tests) are displayed. The significance level is ,050.
a. Significance values have been adjusted by the Bonferroni correction for multiple tests.

Day9

Independent-Samples Kruskal-Wallis Test Summary

Total N	15
Test Statistic	10,900[a]

Degree Of Freedom	4
Asymptotic Sig.(2-sided test)	0.028

a. The test statistic is adjusted for ties.

Pairwise Comparisons of Treatment

Sample 1-Sample 2	Test Statistic	Std. Error	Std. Test Statistic	Sig.	Adj. Sig.[a]
Uncoated-FC	-5.333	3.651	-1.461	0.144	1.000
Uncoated-MC	-6.333	3.651	-1.734	0.083	0.828
Uncoated-MCC	-6.333	3.651	-1.734	0.083	0.828
Uncoated-NC	-12.000	3.651	-3.286	0.001	0.010
FC-MC	-1.000	3.651	-0.274	0.784	1.000
FC-MCC	-1.000	3.651	-0.274	0.784	1.000
FC-NC	-6.667	3.651	-1.826	0.068	0.679
MC-MCC	0.000	3.651	0.000	1.000	1.000
MC-NC	-5.667	3.651	-1.552	0.121	1.000
MCC-NC	-5.667	3.651	-1.552	0.121	1.000

Each row tests the null hypothesis that the Sample 1 and Sample 2 distributions are the same.
Asymptotic significances (2-sided tests) are displayed. The significance level is ,050.
a. Significance values have been adjusted by the Bonferroni correction for multiple tests.

Table S5 Non-parametric a value during 9 days of meat storage*

a*

Day0

Independent-Samples Kruskal-Wallis Test Summary

Total N	15
Test Statistic	7,981[a]
Degree Of Freedom	4
Asymptotic Sig.(2-sided test)	0.092

a. The test statistic is adjusted for ties.

Pairwise Comparisons of Treatment

Sample 1-Sample 2	Test Statistic	Std. Error	Std. Test Statistic	Sig.	Adj. Sig.[a]
NC-MC	1.000	3.648	0.274	0.784	1.000
NC-FC	2.667	3.648	0.731	0.465	1.000
NC-MCC	3.667	3.648	1.005	0.315	1.000
NC-Uncoated	9.333	3.648	2.558	0.011	0.105
MC-FC	1.667	3.648	0.457	0.648	1.000
MC-MCC	-2.667	3.648	-0.731	0.465	1.000
MC-Uncoated	8.333	3.648	2.284	0.022	0.224
FC-MCC	-1.000	3.648	-0.274	0.784	1.000
FC-Uncoated	6.667	3.648	1.827	0.068	0.676
MCC-Uncoated	5.667	3.648	1.553	0.120	1.000

Each row tests the null hypothesis that the Sample 1 and Sample 2 distributions are the same.
Asymptotic significances (2-sided tests) are displayed. The significance level is ,050.
a. Significance values have been adjusted by the Bonferroni correction for multiple tests.

Day 3 a*

Independent-Samples Kruskal-Wallis Test Summary

Total N	15
Test Statistic	12,000[a]
Degree Of Freedom	4
Asymptotic Sig.(2-sided test)	0.017

a. The test statistic is adjusted for ties.

Pairwise Comparisons of Treatment

Sample 1-Sample 2	Test Statistic	Std. Error	Std. Test Statistic	Sig.	Adj. Sig.[a]
MCC-FC	4.000	3.651	1.095	0.273	1.000
MCC-NC	-6.000	3.651	-1.643	0.100	1.000
MCC-MC	8.000	3.651	2.191	0.028	0.285
MCC-Uncoated	12.000	3.651	3.286	0.001	0.010
FC-NC	-2.000	3.651	-0.548	0.584	1.000
FC-MC	-4.000	3.651	-1.095	0.273	1.000
FC-Uncoated	8.000	3.651	2.191	0.028	0.285
NC-MC	2.000	3.651	0.548	0.584	1.000
NC-Uncoated	6.000	3.651	1.643	0.100	1.000
MC-Uncoated	4.000	3.651	1.095	0.273	1.000

Each row tests the null hypothesis that the Sample 1 and Sample 2 distributions are the same.
Asymptotic significances (2-sided tests) are displayed. The significance level is ,050.
a. Significance values have been adjusted by the Bonferroni correction for multiple tests.

Day6

Independent-Samples Kruskal-Wallis Test Summary

Total N	15
Test Statistic	8,433[a]
Degree Of Freedom	4

Asymptotic Sig.(2-sided test)	0.077

a. The test statistic is adjusted for ties.

Pairwise Comparisons of Treatment

Sample 1-Sample 2	Test Statistic	Std. Error	Std. Test Statistic	Sig.	Adj. Sig.[a]
MCC-FC	4.333	3.651	1.187	0.235	1.000
MCC-MC	7.000	3.651	1.917	0.055	0.552
MCC-NC	-7.000	3.651	-1.917	0.055	0.552
MCC-Uncoated	10.000	3.651	2.739	0.006	0.062
FC-MC	-2.667	3.651	-0.730	0.465	1.000
FC-NC	-2.667	3.651	-0.730	0.465	1.000
FC-Uncoated	5.667	3.651	1.552	0.121	1.000
MC-Uncoated	3.000	3.651	0.822	0.411	1.000
NC-Uncoated	3.000	3.651	0.822	0.411	1.000
MC-NC	0.000	3.651	0.000	1.000	1.000

Each row tests the null hypothesis that the Sample 1 and Sample 2 distributions are the same.
Asymptotic significances (2-sided tests) are displayed. The significance level is ,050.
a. Significance values have been adjusted by the Bonferroni correction for multiple tests.

Day9

Independent-Samples Kruskal-Wallis Test Summary

Total N	15
Test Statistic	8,333[a]
Degree Of Freedom	4
Asymptotic Sig.(2-sided test)	0.080

a. The test statistic is adjusted for ties.

Pairwise Comparisons of Treatment

Sample 1-Sample 2	Test Statistic	Std. Error	Std. Test Statistic	Sig.	Adj. Sig.[a]
MC-MCC	-1.333	3.651	-0.365	0.715	1.000

MC-NC	-2.333	3.651	-0.639	0.523	1.000
MC-Uncoated	5.333	3.651	1.461	0.144	1.000
MC-FC	9.333	3.651	2.556	0.011	0.106
MCC-NC	-1.000	3.651	-0.274	0.784	1.000
MCC-Uncoated	4.000	3.651	1.095	0.273	1.000
MCC-FC	8.000	3.651	2.191	0.028	0.285
NC-Uncoated	3.000	3.651	0.822	0.411	1.000
NC-FC	7.000	3.651	1.917	0.055	0.552
Uncoated-FC	-4.000	3.651	-1.095	0.273	1.000

Each row tests the null hypothesis that the Sample 1 and Sample 2 distributions are the same.
Asymptotic significances (2-sided tests) are displayed. The significance level is ,050.
a. Significance values have been adjusted by the Bonferroni correction for multiple tests.

Table S6 Non-parametric b value during 9 days of meat storage*

Day0 b*

Independent-Samples Kruskal-Wallis Test Summary

Total N	60
Test Statistic	7,030[a]
Degree Of Freedom	4
Asymptotic Sig.(2-sided test)	0.134

a. The test statistic is adjusted for ties.

Pairwise Comparisons of Treatment

Sample 1-Sample 2	Test Statistic	Std. Error	Std. Test Statistic	Sig.	Adj. Sig.[a]
MCC-Uncoated	0.417	7.129	0.058	0.953	1.000
MCC-FC	6.792	7.129	0.953	0.341	1.000
MCC-MC	8.375	7.129	1.175	0.240	1.000

MCC-NC	-16.292	7.129	-2.285	0.022	0.223
Uncoated-FC	-6.375	7.129	-0.894	0.371	1.000
Uncoated-MC	-7.958	7.129	-1.116	0.264	1.000
Uncoated-NC	-15.875	7.129	-2.227	0.026	0.260
FC-MC	-1.583	7.129	-0.222	0.824	1.000
FC-NC	-9.500	7.129	-1.333	0.183	1.000
MC-NC	-7.917	7.129	-1.110	0.267	1.000

Each row tests the null hypothesis that the Sample 1 and Sample 2 distributions are the same.
Asymptotic significances (2-sided tests) are displayed. The significance level is ,050.
a. Significance values have been adjusted by the Bonferroni correction for multiple tests.

Day3 b

Independent-Samples Kruskal-Wallis Test Summary

Total N	15
Test Statistic	7,205[a]
Degree Of Freedom	4
Asymptotic Sig.(2-sided test)	0.125

a. The test statistic is adjusted for ties.

Pairwise Comparisons of Treatment

Sample 1-Sample 2	Test Statistic	Std. Error	Std. Test Statistic	Sig.	Adj. Sig.[a]
MCC-MC	3.667	3.648	1.005	0.315	1.000
MCC-Uncoated	3.833	3.648	1.051	0.293	1.000
MCC-FC	4.500	3.648	1.233	0.217	1.000
MCC-NC	-9.667	3.648	-2.650	0.008	0.081
MC-Uncoated	0.167	3.648	0.046	0.964	1.000
MC-FC	0.833	3.648	0.228	0.819	1.000
MC-NC	-6.000	3.648	-1.645	0.100	1.000
Uncoated-FC	-0.667	3.648	-0.183	0.855	1.000

Uncoated-NC	-5.833	3.648	-1.599	0.110	1.000
FC-NC	-5.167	3.648	-1.416	0.157	1.000

Each row tests the null hypothesis that the Sample 1 and Sample 2 distributions are the same.
Asymptotic significances (2-sided tests) are displayed. The significance level is ,050.
a. Significance values have been adjusted by the Bonferroni correction for multiple tests.

Day6 b

Independent-Samples Kruskal-Wallis Test Summary

Total N	15
Test Statistic	4,516[a]
Degree Of Freedom	4
Asymptotic Sig.(2-sided test)	0.341

a. The test statistic is adjusted for ties.

Pairwise Comparisons of Treatment

Sample 1-Sample 2	Test Statistic	Std. Error	Std. Test Statistic	Sig.	Adj. Sig.[a]
MCC-MC	0.833	3.648	0.228	0.819	1.000
MCC-FC	2.333	3.648	0.640	0.522	1.000
MCC-Uncoated	4.167	3.648	1.142	0.253	1.000
MCC-NC	-6.833	3.648	-1.873	0.061	0.611
MC-FC	1.500	3.648	0.411	0.681	1.000
MC-Uncoated	3.333	3.648	0.914	0.361	1.000
MC-NC	-6.000	3.648	-1.645	0.100	1.000
FC-Uncoated	1.833	3.648	0.503	0.615	1.000
FC-NC	-4.500	3.648	-1.233	0.217	1.000
Uncoated-NC	-2.667	3.648	-0.731	0.465	1.000

Each row tests the null hypothesis that the Sample 1 and Sample 2 distributions are the same.
Asymptotic significances (2-sided tests) are displayed. The significance level is ,050.
a. Significance values have been adjusted by the Bonferroni correction for multiple tests.

Day9 b

Independent-Samples Kruskal-Wallis Test Summary

Total N	15
Test Statistic	4,767[a]
Degree Of Freedom	4
Asymptotic Sig.(2-sided test)	0.312

a. The test statistic is adjusted for ties.

Pairwise Comparisons of Treatment

Sample 1-Sample 2	Test Statistic	Std. Error	Std. Test Statistic	Sig.	Adj. Sig.[a]
Uncoated-MCC	-1.667	3.651	-0.456	0.648	1.000
Uncoated-FC	-3.000	3.651	-0.822	0.411	1.000
Uncoated-NC	-4.667	3.651	-1.278	0.201	1.000
Uncoated-MC	-7.333	3.651	-2.008	0.045	0.446
MCC-FC	1.333	3.651	0.365	0.715	1.000
MCC-NC	-3.000	3.651	-0.822	0.411	1.000
MCC-MC	5.667	3.651	1.552	0.121	1.000
FC-NC	-1.667	3.651	-0.456	0.648	1.000
FC-MC	-4.333	3.651	-1.187	0.235	1.000
NC-MC	2.667	3.651	0.730	0.465	1.000

Each row tests the null hypothesis that the Sample 1 and Sample 2 distributions are the same.
Asymptotic significances (2-sided tests) are displayed. The significance level is ,050.
a. Significance values have been adjusted by the Bonferroni correction for multiple tests.

Table S7 Non-parametric in mesophilic analysis of stored fresh pork minced meat for 9 days

Nonparametric Tests

Hypothesis Test Summary

	Null Hypothesis	Test	Sig.[a,b]	Decision
1	The distribution of DAY_0 is the same across categories of Samples.	Independent-Samples Kruskal-Wallis Test	1.000	Retain the null hypothesis.
2	The distribution of DAY_3 is the same across categories of Samples.	Independent-Samples Kruskal-Wallis Test	0.012	Reject the null hypothesis.
3	The distribution of DAY_6 is the same across categories of Samples.	Independent-Samples Kruskal-Wallis Test	0.012	Reject the null hypothesis.
4	The distribution of DAY_9 is the same across categories of Samples.	Independent-Samples Kruskal-Wallis Test	0.012	Reject the null hypothesis.

a. The significance level is ,050.

b. Asymptotic significance is displayed.

DAY_0 across Samples

Independent-Samples Kruskal-Wallis Test Summary

Total N	15
Test Statistic	,000[a]
Degree Of Freedom	4

Asymptotic Sig.(2-sided test)	1.000

a. The test statistic is adjusted for ties.

Pairwise Comparisons of Samples

Sample 1-Sample 2	Test Statistic	Std. Error	Std. Test Statistic	Sig.	Adj. Sig.[a]
Uncoated-FC	0.000	3.450	0.000	1.000	1.000
Uncoated-MC	0.000	3.450	0.000	1.000	1.000
Uncoated-MCC	0.000	3.450	0.000	1.000	1.000
Uncoated-NC	0.000	3.450	0.000	1.000	1.000
FC-MC	0.000	3.450	0.000	1.000	1.000
FC-MCC	0.000	3.450	0.000	1.000	1.000
FC-NC	0.000	3.450	0.000	1.000	1.000
MC-MCC	0.000	3.450	0.000	1.000	1.000
MC-NC	0.000	3.450	0.000	1.000	1.000
MCC-NC	0.000	3.450	0.000	1.000	1.000

Each row tests the null hypothesis that the Sample 1 and Sample 2 distributions are the same.
Asymptotic significances (2-sided tests) are displayed. The significance level is ,050.

a. Significance values have been adjusted by the Bonferroni correction for multiple tests.

DAY_3 across Samples

Independent-Samples Kruskal-Wallis Test Summary

Total N	15
Test Statistic	12,900[a]
Degree Of Freedom	4
Asymptotic Sig.(2-sided test)	0.012

a. The test statistic is adjusted for ties.

Pairwise Comparisons of Samples

Sample 1-Sample 2	Test Statistic	Std. Error	Std. Test Statistic	Sig.	Adj. Sig.[a]
NC-MCC	3.000	3.651	0.822	0.411	1.000
NC-MC	7.000	3.651	1.917	0.055	0.552
NC-FC	8.000	3.651	2.191	0.028	0.285
NC-Uncoated	12.000	3.651	3.286	0.001	0.010
MCC-MC	4.000	3.651	1.095	0.273	1.000

MCC-FC	5.000	3.651	1.369	0.171	1.000
MCC-Uncoated	9.000	3.651	2.465	0.014	0.137
MC-FC	1.000	3.651	0.274	0.784	1.000
MC-Uncoated	5.000	3.651	1.369	0.171	1.000
FC-Uncoated	4.000	3.651	1.095	0.273	1.000

Each row tests the null hypothesis that the Sample 1 and Sample 2 distributions are the same.
Asymptotic significances (2-sided tests) are displayed. The significance level is ,050.

a. Significance values have been adjusted by the Bonferroni correction for multiple tests.

DAY_6 across Samples

Independent-Samples Kruskal-Wallis Test Summary

Total N	15
Test Statistic	12,928[a]
Degree Of Freedom	4
Asymptotic Sig.(2-sided test)	0.012

a. The test statistic is adjusted for ties.

Pairwise Comparisons of Samples

Sample 1-Sample 2	Test Statistic	Std. Error	Std. Test Statistic	Sig.	Adj. Sig.[a]
NC-MCC	3.167	3.642	0.870	0.385	1.000
NC-FC	6.500	3.642	1.785	0.074	0.743
NC-MC	8.333	3.642	2.288	0.022	0.221
NC-Uncoated	12.000	3.642	3.295	0.001	0.010
MCC-FC	3.333	3.642	0.915	0.360	1.000
MCC-MC	5.167	3.642	1.419	0.156	1.000
MCC-Uncoated	8.833	3.642	2.426	0.015	0.153
FC-MC	-1.833	3.642	-0.503	0.615	1.000
FC-Uncoated	5.500	3.642	1.510	0.131	1.000
MC-Uncoated	3.667	3.642	1.007	0.314	1.000

Each row tests the null hypothesis that the Sample 1 and Sample 2 distributions are the same.
Asymptotic significances (2-sided tests) are displayed. The significance level is ,050.

a. Significance values have been adjusted by the Bonferroni correction for multiple tests.

DAY_9 across Samples

Independent-Samples Kruskal-Wallis Test Summary

Total N	15
Test Statistic	12,928[a]

Degree Of Freedom	4
Asymptotic Sig.(2-sided test)	0.012

a. The test statistic is adjusted for ties.

Pairwise Comparisons of Samples

Sample 1-Sample 2	Test Statistic	Std. Error	Std. Test Statistic	Sig.	Adj. Sig.[a]
NC-MCC	3.167	3.642	0.870	0.385	1.000
NC-FC	6.500	3.642	1.785	0.074	0.743
NC-MC	8.333	3.642	2.288	0.022	0.221
NC-Uncoated	12.000	3.642	3.295	0.001	0.010
MCC-FC	3.333	3.642	0.915	0.360	1.000
MCC-MC	5.167	3.642	1.419	0.156	1.000
MCC-Uncoated	8.833	3.642	2.426	0.015	0.153
FC-MC	-1.833	3.642	-0.503	0.615	1.000
FC-Uncoated	5.500	3.642	1.510	0.131	1.000
MC-Uncoated	3.667	3.642	1.007	0.314	1.000

Each row tests the null hypothesis that the Sample 1 and Sample 2 distributions are the same.
Asymptotic significances (2-sided tests) are displayed. The significance level is ,050.
a. Significance values have been adjusted by the Bonferroni correction for multiple tests.

Table S8 LAB bacteria microbiological analysis in pork minced meat

LAB Nonparametric Tests

Hypothesis Test Summary

	Null Hypothesis	Test	Sig.a,b	Decision
1	The distribution of DAY_0 is the same across categories of Samples.	Independent-Samples Kruskal-Wallis Test	1,000	Retain the null hypothesis.

2	The distribution of DAY_3 is the same across categories of Samples.	Independent-Samples Kruskal-Wallis Test	,015	Reject the null hypothesis.
3	The distribution of DAY_6 is the same across categories of Samples.	Independent-Samples Kruskal-Wallis Test	,011	Reject the null hypothesis.
4	The distribution of DAY_9 is the same across categories of Samples.	Independent-Samples Kruskal-Wallis Test	,010	Reject the null hypothesis.

a The significance level is ,050.

b Asymptotic significance is displayed.

DAY_0 across Samples

Independent-Samples Kruskal-Wallis Test Summary

Total N	15
Test Statistic	,000a
Degree Of Freedom	4
Asymptotic Sig.(2-sided test)	1,000

a The test statistic is adjusted for ties.

Pairwise Comparisons of Samples

Sample 1-Sample 2	Test Statistic	Std. Error	Std. Test Statistic	Sig.	Adj. Sig.[a]
Uncoated-FC	0.000	3.450	0.000	1.000	1.000
Uncoated-MC	0.000	3.450	0.000	1.000	1.000
Uncoated-MCC	0.000	3.450	0.000	1.000	1.000
Uncoated-NC	0.000	3.450	0.000	1.000	1.000
FC-MC	0.000	3.450	0.000	1.000	1.000
FC-MCC	0.000	3.450	0.000	1.000	1.000

FC-NC	0.000	3.450	0.000	1.000	1.000
MC-MCC	0.000	3.450	0.000	1.000	1.000
MC-NC	0.000	3.450	0.000	1.000	1.000
MCC-NC	0.000	3.450	0.000	1.000	1.000

Each row tests the null hypothesis that the Sample 1 and Sample 2 distributions are the same.
Asymptotic significances (2-sided tests) are displayed. The significance level is ,050.
a. Significance values have been adjusted by the Bonferroni correction for multiple tests.

DAY_3 across Samples

Independent-Samples Kruskal-Wallis Test Summary

Total N	15
Test Statistic	12,341[a]
Degree Of Freedom	4

Asymptotic Sig.(2-sided test)	0.015

a. The test statistic is adjusted for ties.

Pairwise Comparisons of Samples

Sample 1-Sample 2	Test Statistic	Std. Error	Std. Test Statistic	Sig.	Adj. Sig.[a]
NC-MCC	3.000	3.632	0.826	0.409	1.000
NC-MC	6.833	3.632	1.881	0.060	0.599
NC-FC	9.000	3.632	2.478	0.013	0.132
NC-Uncoated	11.167	3.632	3.075	0.002	0.021
MCC-MC	3.833	3.632	1.055	0.291	1.000
MCC-FC	6.000	3.632	1.652	0.099	0.985
MCC-Uncoated	8.167	3.632	2.249	0.025	0.245
MC-FC	2.167	3.632	0.597	0.551	1.000
MC-Uncoated	4.333	3.632	1.193	0.233	1.000
FC-Uncoated	2.167	3.632	0.597	0.551	1.000

Each row tests the null hypothesis that the Sample 1 and Sample 2 distributions are the same.
Asymptotic significances (2-sided tests) are displayed. The significance level is ,050.
a. Significance values have been adjusted by the Bonferroni correction for multiple tests.

DAY_6 across Samples

Independent-Samples Kruskal-Wallis Test Summary

Total N	15
Test Statistic	13,080[a]
Degree Of Freedom	4
Asymptotic Sig.(2-sided test)	0.011

a. The test statistic is adjusted for ties.

Pairwise Comparisons of Samples

Sample 1-Sample 2	Test Statistic	Std. Error	Std. Test Statistic	Sig.	Adj. Sig.[a]
NC-MCC	3.000	3.645	0.823	0.410	1.000
NC-FC	6.667	3.645	1.829	0.067	0.674
NC-MC	8.333	3.645	2.286	0.022	0.222
NC-Uncoated	12.000	3.645	3.292	0.001	0.010
MCC-FC	3.667	3.645	1.006	0.314	1.000
MCC-MC	5.333	3.645	1.463	0.143	1.000
MCC-Uncoated	9.000	3.645	2.469	0.014	0.135

FC-MC	-1.667	3.645	-0.457	0.647	1.000
FC-Uncoated	5.333	3.645	1.463	0.143	1.000
MC-Uncoated	3.667	3.645	1.006	0.314	1.000

Each row tests the null hypothesis that the Sample 1 and Sample 2 distributions are the same.
Asymptotic significances (2-sided tests) are displayed. The significance level is ,050.
a. Significance values have been adjusted by the Bonferroni correction for multiple tests.

DAY_9 across Samples

Independent-Samples Kruskal-Wallis Test Summary

Total N	15
Test Statistic	13,382[a]
Degree Of Freedom	4
Asymptotic Sig.(2-sided test)	0.010

a. The test statistic is adjusted for ties.

Pairwise Comparisons of Samples

Sample 1-Sample 2	Test Statistic	Std. Error	Std. Test Statistic	Sig.	Adj. Sig.[a]
NC-MCC	3.000	3.648	0.822	0.411	1.000

NC-MC	6.167	3.648	1.690	0.091	0.910
NC-FC	8.833	3.648	2.421	0.015	0.155
NC-Uncoated	12.000	3.648	3.289	0.001	0.010
MCC-MC	3.167	3.648	0.868	0.385	1.000
MCC-FC	5.833	3.648	1.599	0.110	1.000
MCC-Uncoated	9.000	3.648	2.467	0.014	0.136
MC-FC	2.667	3.648	0.731	0.465	1.000
MC-Uncoated	5.833	3.648	1.599	0.110	1.000
FC-Uncoated	3.167	3.648	0.868	0.385	1.000

Each row tests the null hypothesis that the Sample 1 and Sample 2 distributions are the same.
Asymptotic significances (2-sided tests) are displayed. The significance level is ,050.
a. Significance values have been adjusted by the Bonferroni correction for multiple tests.

Table S9 Psychrotrophic bacteria microbiological analysis in pork minced meat

Nonparametric Tests

Hypothesis Test Summary

	Null Hypothesis	Test	Sig.[a,b]	Decision
1	The distribution of DAY_0 is the same across categories of Samples.	Independent-Samples Kruskal-Wallis Test	1.000	Retain the null hypothesis.
2	The distribution of DAY_3 is the same across	Independent-Samples Kruskal-Wallis Test	0.014	Reject the null hypothesis.

	categories of Samples.			
3	The distribution of DAY_6 is the same across categories of Samples.	Independent-Samples Kruskal-Wallis Test	0.013	Reject the null hypothesis.
4	The distribution of DAY_9 is the same across categories of Samples.	Independent-Samples Kruskal-Wallis Test	0.010	Reject the null hypothesis.

a. The significance level is ,050.

b. Asymptotic significance is displayed.

DAY_0 across Samples

Independent-Samples Kruskal-Wallis Test Summary	
Total N	15
Test Statistic	,000a
Degree Of Freedom	4
Asymptotic Sig.(2-sided test)	1,000

a The test statistic is adjusted for ties.

Pairwise Comparisons of Samples

Sample 1-Sample 2	Test Statistic	Std. Error	Std. Test Statistic	Sig.	Adj. Sig.[a]
Uncoated-FC	0.000	3.450	0.000	1.000	1.000
Uncoated-MC	0.000	3.450	0.000	1.000	1.000
Uncoated-MCC	0.000	3.450	0.000	1.000	1.000

Uncoated-NC	0.000	3.450	0.000	1.000	1.000
FC-MC	0.000	3.450	0.000	1.000	1.000
FC-MCC	0.000	3.450	0.000	1.000	1.000
FC-NC	0.000	3.450	0.000	1.000	1.000
MC-MCC	0.000	3.450	0.000	1.000	1.000
MC-NC	0.000	3.450	0.000	1.000	1.000
MCC-NC	0.000	3.450	0.000	1.000	1.000

Each row tests the null hypothesis that the Sample 1 and Sample 2 distributions are the same.
Asymptotic significances (2-sided tests) are displayed. The significance level is ,050.
a. Significance values have been adjusted by the Bonferroni correction for multiple tests.

DAY_3 across Samples

Independent-Samples Kruskal-Wallis Test Summary

Total N	15
Test Statistic	12,534[a]
Degree Of Freedom	4
Asymptotic Sig.(2-sided test)	0.01379

a. The test statistic is adjusted for ties.

Pairwise Comparisons of Samples

Sample 1-Sample 2	Test Statistic	Std. Error	Std. Test Statistic	Sig.	Adj. Sig.[a]
NC-MCC	3.333	3.632	0.918	0.359	1.000
NC-FC	7.333	3.632	2.019	0.043	0.435
NC-MC	7.333	3.632	2.019	0.043	0.435
NC-Uncoated	12.000	3.632	3.304	0.001	0.010
MCC-FC	4.000	3.632	1.101	0.271	1.000
MCC-MC	4.000	3.632	1.101	0.271	1.000

MCC-Uncoated	8.667	3.632	2.386	0.017	0.170
FC-Uncoated	4.667	3.632	1.285	0.199	1.000
MC-Uncoated	4.667	3.632	1.285	0.199	1.000
FC-MC	0.000	3.632	0.000	1.000	1.000

Each row tests the null hypothesis that the Sample 1 and Sample 2 distributions are the same.
Asymptotic significances (2-sided tests) are displayed. The significance level is ,050.
a. Significance values have been adjusted by the Bonferroni correction for multiple tests.

DAY_6 across Samples

Independent-Samples Kruskal-Wallis Test Summary

Total N	15
Test Statistic	12,657[a]
Degree Of Freedom	4
Asymptotic Sig.(2-sided test)	0.013

a. The test statistic is adjusted for ties.

Pairwise Comparisons of Samples

Sample 1-Sample 2	Test Statistic	Std. Error	Std. Test Statistic	Sig.	Adj. Sig.[a]
NC-MCC	1.667	3.638	0.458	0.647	1.000
NC-MC	6.000	3.638	1.649	0.099	0.991
NC-FC	7.667	3.638	2.107	0.035	0.351

NC-Uncoated	11.333	3.638	3.115	0.002	0.018
MCC-MC	4.333	3.638	1.191	0.234	1.000
MCC-FC	6.000	3.638	1.649	0.099	0.991
MCC-Uncoated	9.667	3.638	2.657	0.008	0.079
MC-FC	1.667	3.638	0.458	0.647	1.000
MC-Uncoated	5.333	3.638	1.466	0.143	1.000
FC-Uncoated	3.667	3.638	1.008	0.314	1.000

Each row tests the null hypothesis that the Sample 1 and Sample 2 distributions are the same.
Asymptotic significances (2-sided tests) are displayed. The significance level is ,050.
a. Significance values have been adjusted by the Bonferroni correction for multiple tests.

DAY_9 across Samples

Independent-Samples Kruskal-Wallis Test Summary

Total N	15
Test Statistic	13,382[a]
Degree Of Freedom	4
Asymptotic Sig.(2-sided test)	0.010

a. The test statistic is adjusted for ties.

Pairwise Comparisons of Samples

Sample 1-Sample 2	Test Statistic	Std. Error	Std. Test Statistic	Sig.	Adj. Sig.[a]
NC-MCC	3.000	3.648	0.822	0.411	1.000
NC-MC	6.167	3.648	1.690	0.091	0.910
NC-FC	8.833	3.648	2.421	0.015	0.155
NC-Uncoated	12.000	3.648	3.289	0.001	0.010
MCC-MC	3.167	3.648	0.868	0.385	1.000
MCC-FC	5.833	3.648	1.599	0.110	1.000
MCC-Uncoated	9.000	3.648	2.467	0.014	0.136

MC-FC	2.667	3.648	0.731	0.465	1.000
MC-Uncoated	5.833	3.648	1.599	0.110	1.000
FC-Uncoated	3.167	3.648	0.868	0.385	1.000

Each row tests the null hypothesis that the Sample 1 and Sample 2 distributions are the same.
Asymptotic significances (2-sided tests) are displayed. The significance level is ,050.
a. Significance values have been adjusted by the Bonferroni correction for multiple tests.

Table S10 Non-parametrics of Sensory test

Hypothesis Test Summary

	Null Hypothesis	Test	Sig.[a,b]	Decision
1	The distribution of Results_Appearence is the same across categories of Treatment.	Independent-Samples Kruskal-Wallis Test	0.000	Reject the null hypothesis.
2	The distribution of Results_Odor is the same across categories of Treatment.	Independent-Samples Kruskal-Wallis Test	0.000	Reject the null hypothesis.
3	The distribution of Results_Colour is the same across categories of Treatment.	Independent-Samples Kruskal-Wallis Test	0.000	Reject the null hypothesis.
4	The distribution of Results_Texture is the same across categories of Treatment.	Independent-Samples Kruskal-Wallis Test	0.709	Retain the null hypothesis.

a. The significance level is ,050.

b. Asymptotic significance is displayed.

Pairwise Comparisons of Treatment - Appearance

Sample 1-Sample 2	Test Statistic	Std. Error	Std. Test Statistic	Sig.	Adj. Sig.[a]
NC-MCC	46.783	15.639	2.992	0.003	0.028
NC-MC	75.317	15.639	4.816	0.000	0.000
NC-FC	121.783	15.639	7.787	0.000	0.000
NC-Uncoated	190.117	15.639	12.157	0.000	0.000
MCC-MC	28.533	15.639	1.825	0.068	0.681
MCC-FC	75.000	15.639	4.796	0.000	0.000
MCC-Uncoated	143.333	15.639	9.165	0.000	0.000
MC-FC	46.467	15.639	2.971	0.003	0.030
MC-Uncoated	114.800	15.639	7.341	0.000	0.000
FC-Uncoated	68.333	15.639	4.370	0.000	0.000

Each row tests the null hypothesis that the Sample 1 and Sample 2 distributions are the same.
Asymptotic significances (2-sided tests) are displayed. The significance level is ,050.
a. Significance values have been adjusted by the Bonferroni correction for multiple tests.

Pairwise Comparisons of Treatment - Colour

Sample 1-Sample 2	Test Statistic	Std. Error	Std. Test Statistic	Sig.	Adj. Sig.[a]
NC-MCC	47.183	15.496	3.045	0.002	0.023
NC-MC	84.083	15.496	5.426	0.000	0.000
NC-FC	112.600	15.496	7.266	0.000	0.000
NC-Uncoated	172.342	15.496	11.121	0.000	0.000
MCC-MC	36.900	15.496	2.381	0.017	0.173
MCC-FC	65.417	15.496	4.221	0.000	0.000
MCC-Uncoated	125.158	15.496	8.077	0.000	0.000
MC-FC	28.517	15.496	1.840	0.066	0.657
MC-Uncoated	88.258	15.496	5.695	0.000	0.000
FC-Uncoated	59.742	15.496	3.855	0.000	0.001

Each row tests the null hypothesis that the Sample 1 and Sample 2 distributions are the same.
Asymptotic significances (2-sided tests) are displayed. The significance level is ,050.

a. Significance values have been adjusted by the Bonferroni correction for multiple tests.

Pairwise Comparisons of Treatment - Odor

Sample 1-Sample 2	Test Statistic	Std. Error	Std. Test Statistic	Sig.	Adj. Sig.[a]
NC-MCC	63.008	15.633	4.031	0.000	0.001
NC-MC	109.317	15.633	6.993	0.000	0.000
NC-FC	146.392	15.633	9.365	0.000	0.000
NC-Uncoated	168.325	15.633	10.768	0.000	0.000
MCC-MC	46.308	15.633	2.962	0.003	0.031
MCC-FC	83.383	15.633	5.334	0.000	0.000
MCC-Uncoated	105.317	15.633	6.737	0.000	0.000
MC-FC	37.075	15.633	2.372	0.018	0.177
MC-Uncoated	59.008	15.633	3.775	0.000	0.002
FC-Uncoated	21.933	15.633	1.403	0.161	1.000

Each row tests the null hypothesis that the Sample 1 and Sample 2 distributions are the same.
Asymptotic significances (2-sided tests) are displayed. The significance level is ,050.
a. Significance values have been adjusted by the Bonferroni correction for multiple tests.

Appearance – Day 0

Pairwise Comparisons of Treatment_day

Sample 1-Sample 2	Test Statistic	Std. Error	Std. Test Statistic	Sig.	Adj. Sig.[a]
MC-NC	-2.500	6.617	-0.378	0.706	1.000
MC-MCC	-10.000	6.617	-1.511	0.131	1.000
MC-FC	15.000	6.617	2.267	0.023	0.234

MC-Uncoated	17.500	6.617	2.645	0.008	0.082
NC-MCC	7.500	6.617	1.133	0.257	1.000
NC-FC	12.500	6.617	1.889	0.059	0.589
NC-Uncoated	15.000	6.617	2.267	0.023	0.234
MCC-FC	5.000	6.617	0.756	0.450	1.000
MCC-Uncoated	7.500	6.617	1.133	0.257	1.000
FC-Uncoated	2.500	6.617	0.378	0.706	1.000

Each row tests the null hypothesis that the Sample 1 and Sample 2 distributions are the same.
Asymptotic significances (2-sided tests) are displayed. The significance level is ,050.
a. Significance values have been adjusted by the Bonferroni correction for multiple tests.

Appearance Day 3

Pairwise Comparisons of Treatment_day

Sample 1-Sample 2	Test Statistic	Std. Error	Std. Test Statistic	Sig.	Adj. Sig.[a]
Uncoated-FC	-4.600	7.465	-0.616	0.538	1.000
Uncoated-MC	-13.067	7.465	-1.750	0.080	0.801
Uncoated-NC	-22.933	7.465	-3.072	0.002	0.021
Uncoated-MCC	-28.733	7.465	-3.849	0.000	0.001

FC-MC	-8.467	7.465	-1.134	0.257	1.000
FC-NC	-18.333	7.465	-2.456	0.014	0.141
FC-MCC	-24.133	7.465	-3.233	0.001	0.012
MC-NC	-9.867	7.465	-1.322	0.186	1.000
MC-MCC	-15.667	7.465	-2.099	0.036	0.359
NC-MCC	5.800	7.465	0.777	0.437	1.000

Each row tests the null hypothesis that the Sample 1 and Sample 2 distributions are the same.
Asymptotic significances (2-sided tests) are displayed. The significance level is ,050.
a. Significance values have been adjusted by the Bonferroni correction for multiple tests.

Appearance Day 6

Pairwise Comparisons of Treatment_day

Sample 1-Sample 2	Test Statistic	Std. Error	Std. Test Statistic	Sig.	Adj. Sig.[a]
Uncoated-FC	-7.333	7.770	-0.944	0.345	1.000
Uncoated-MC	-22.567	7.770	-2.904	0.004	0.037
Uncoated-NC	-38.033	7.770	-4.895	0.000	0.000
Uncoated-MCC	-42.567	7.770	-5.479	0.000	0.000
FC-MC	-15.233	7.770	-1.961	0.050	0.499
FC-NC	-30.700	7.770	-3.951	0.000	0.001
FC-MCC	-35.233	7.770	-4.535	0.000	0.000
MC-NC	-15.467	7.770	-1.991	0.047	0.465

MC-MCC	-20.000	7.770	-2.574	0.010	0.100
NC-MCC	4.533	7.770	0.583	0.560	1.000

Each row tests the null hypothesis that the Sample 1 and Sample 2 distributions are the same.
Asymptotic significances (2-sided tests) are displayed. The significance level is ,050.
a. Significance values have been adjusted by the Bonferroni correction for multiple tests.

Appearance Day 9

Pairwise Comparisons of Treatment_day

Sample 1-Sample 2	Test Statistic	Std. Error	Std. Test Statistic	Sig.	Adj. Sig.[a]
Uncoated-FC	-4.200	7.661	-0.548	0.584	1.000
Uncoated-MC	-30.467	7.661	-3.977	0.000	0.001
Uncoated-NC	-39.100	7.661	-5.104	0.000	0.000
Uncoated-MCC	-49.233	7.661	-6.427	0.000	0.000
FC-MC	-26.267	7.661	-3.429	0.001	0.006
FC-NC	-34.900	7.661	-4.556	0.000	0.000
FC-MCC	-45.033	7.661	-5.879	0.000	0.000
MC-NC	-8.633	7.661	-1.127	0.260	1.000
MC-MCC	-18.767	7.661	-2.450	0.014	0.143
NC-MCC	10.133	7.661	1.323	0.186	1.000

Each row tests the null hypothesis that the Sample 1 and Sample 2 distributions are the same.
Asymptotic significances (2-sided tests) are displayed. The significance level is ,050.
a. Significance values have been adjusted by the Bonferroni correction for multiple tests.

Odor Day 0

Pairwise Comparisons of Treatment_day

Sample 1-Sample 2	Test Statistic	Std. Error	Std. Test Statistic	Sig.	Adj. Sig.[a]
FC-MC	-4.967	7.376	-0.673	0.501	1.000
FC-MCC	-7.000	7.376	-0.949	0.343	1.000
FC-Uncoated	21.000	7.376	2.847	0.004	0.044
FC-NC	-23.033	7.376	-3.123	0.002	0.018
MC-MCC	-2.033	7.376	-0.276	0.783	1.000
MC-Uncoated	16.033	7.376	2.174	0.030	0.297
MC-NC	-18.067	7.376	-2.449	0.014	0.143
MCC-Uncoated	14.000	7.376	1.898	0.058	0.577
MCC-NC	-16.033	7.376	-2.174	0.030	0.297
Uncoated-NC	-2.033	7.376	-0.276	0.783	1.000

Each row tests the null hypothesis that the Sample 1 and Sample 2 distributions are the same.
Asymptotic significances (2-sided tests) are displayed. The significance level is ,050.
a. Significance values have been adjusted by the Bonferroni correction for multiple tests.

Day_3_Odor across Treatment_day

Pairwise Comparisons of Treatment_day

Sample 1-Sample 2	Test Statistic	Std. Error	Std. Test Statistic	Sig.	Adj. Sig.[a]
FC-MC	-20.700	7.628	-2.714	0.007	0.067
FC-Uncoated	34.100	7.628	4.470	0.000	0.000
FC-MCC	-35.633	7.628	-4.671	0.000	0.000
FC-NC	-45.567	7.628	-5.973	0.000	0.000
MC-Uncoated	13.400	7.628	1.757	0.079	0.790
MC-MCC	-14.933	7.628	-1.958	0.050	0.503
MC-NC	-24.867	7.628	-3.260	0.001	0.011
Uncoated-MCC	-1.533	7.628	-0.201	0.841	1.000
Uncoated-NC	-11.467	7.628	-1.503	0.133	1.000
MCC-NC	-9.933	7.628	-1.302	0.193	1.000

Each row tests the null hypothesis that the Sample 1 and Sample 2 distributions are the same.
Asymptotic significances (2-sided tests) are displayed. The significance level is ,050.
a. Significance values have been adjusted by the Bonferroni correction for multiple tests.

Day_6_Odor across Treatment_day

Pairwise Comparisons of Treatment_day

Sample 1-Sample 2	Test Statistic	Std. Error	Std. Test Statistic	Sig.	Adj. Sig.[a]
Uncoated-FC	-1.533	7.613	-0.201	0.840	1.000
Uncoated-MC	-24.700	7.613	-3.244	0.001	0.012

Uncoated-MCC	-30.967	7.613	-4.068	0.000	0.000
Uncoated-NC	-47.133	7.613	-6.191	0.000	0.000
FC-MC	-23.167	7.613	-3.043	0.002	0.023
FC-MCC	-29.433	7.613	-3.866	0.000	0.001
FC-NC	-45.600	7.613	-5.990	0.000	0.000
MC-MCC	-6.267	7.613	-0.823	0.410	1.000
MC-NC	-22.433	7.613	-2.947	0.003	0.032
MCC-NC	-16.167	7.613	-2.124	0.034	0.337

Each row tests the null hypothesis that the Sample 1 and Sample 2 distributions are the same.
Asymptotic significances (2-sided tests) are displayed. The significance level is ,050.
a. Significance values have been adjusted by the Bonferroni correction for multiple tests.

Day_9_Odor across Treatment_day

Pairwise Comparisons of Treatment_day

Sample 1-Sample 2	Test Statistic	Std. Error	Std. Test Statistic	Sig.	Adj. Sig.[a]
Uncoated-FC	-1.333	7.773	-0.172	0.864	1.000
Uncoated-MC	-15.033	7.773	-1.934	0.053	0.531
Uncoated-MCC	-28.600	7.773	-3.680	0.000	0.002
Uncoated-NC	-46.700	7.773	-6.008	0.000	0.000
FC-MC	-13.700	7.773	-1.763	0.078	0.780
FC-MCC	-27.267	7.773	-3.508	0.000	0.005

FC-NC	-45.367	7.773	-5.837	0.000	0.000
MC-MCC	-13.567	7.773	-1.745	0.081	0.809
MC-NC	-31.667	7.773	-4.074	0.000	0.000
MCC-NC	-18.100	7.773	-2.329	0.020	0.199

Each row tests the null hypothesis that the Sample 1 and Sample 2 distributions are the same.
Asymptotic significances (2-sided tests) are displayed. The significance level is ,050.
a. Significance values have been adjusted by the Bonferroni correction for multiple tests.

Day_0_Texture across Treatment_day

Pairwise Comparisons of Treatment_day

Sample 1-Sample 2	Test Statistic	Std. Error	Std. Test Statistic	Sig.	Adj. Sig.[a]
FC-MC	-2.500	6.357	-0.393	0.694	1.000
FC-Uncoated	5.000	6.357	0.787	0.432	1.000
FC-MCC	-5.000	6.357	-0.787	0.432	1.000
FC-NC	-5.000	6.357	-0.787	0.432	1.000
MC-Uncoated	2.500	6.357	0.393	0.694	1.000
MC-MCC	-2.500	6.357	-0.393	0.694	1.000
MC-NC	-2.500	6.357	-0.393	0.694	1.000
Uncoated-MCC	0.000	6.357	0.000	1.000	1.000
Uncoated-NC	0.000	6.357	0.000	1.000	1.000
MCC-NC	0.000	6.357	0.000	1.000	1.000

Each row tests the null hypothesis that the Sample 1 and Sample 2 distributions are the same.
Asymptotic significances (2-sided tests) are displayed. The significance level is ,050.

a. Significance values have been adjusted by the Bonferroni correction for multiple tests.

Day_3_Texture across Treatment_day

Pairwise Comparisons of Treatment_day

Sample 1-Sample 2	Test Statistic	Std. Error	Std. Test Statistic	Sig.	Adj. Sig.[a]
FC-Uncoated	4.333	7.187	0.603	0.547	1.000
FC-MC	-7.600	7.187	-1.058	0.290	1.000
FC-NC	-14.667	7.187	-2.041	0.041	0.413
FC-MCC	-20.067	7.187	-2.792	0.005	0.052
Uncoated-MC	-3.267	7.187	-0.455	0.649	1.000
Uncoated-NC	-10.333	7.187	-1.438	0.150	1.000
Uncoated-MCC	-15.733	7.187	-2.189	0.029	0.286
MC-NC	-7.067	7.187	-0.983	0.325	1.000
MC-MCC	-12.467	7.187	-1.735	0.083	0.828
NC-MCC	5.400	7.187	0.751	0.452	1.000

Each row tests the null hypothesis that the Sample 1 and Sample 2 distributions are the same.
Asymptotic significances (2-sided tests) are displayed. The significance level is ,050.
a. Significance values have been adjusted by the Bonferroni correction for multiple tests.

Day_6_Texture across Treatment_day

Pairwise Comparisons of Treatment_day

Sample 1-Sample 2	Test Statistic	Std. Error	Std. Test Statistic	Sig.	Adj. Sig.[a]
FC-Uncoated	13.267	7.706	1.722	0.085	0.851
FC-NC	-26.067	7.706	-3.383	0.001	0.007
FC-MC	-31.367	7.706	-4.071	0.000	0.000
FC-MCC	-46.967	7.706	-6.095	0.000	0.000
Uncoated-NC	-12.800	7.706	-1.661	0.097	0.967
Uncoated-MC	-18.100	7.706	-2.349	0.019	0.188
Uncoated-MCC	-33.700	7.706	-4.373	0.000	0.000
NC-MC	5.300	7.706	0.688	0.492	1.000
NC-MCC	20.900	7.706	2.712	0.007	0.067
MC-MCC	-15.600	7.706	-2.024	0.043	0.429

Each row tests the null hypothesis that the Sample 1 and Sample 2 distributions are the same.
Asymptotic significances (2-sided tests) are displayed. The significance level is ,050.
a. Significance values have been adjusted by the Bonferroni correction for multiple tests.

Day_9_Texture across Treatment_day

Pairwise Comparisons of Treatment_day

Sample 1-Sample 2	Test Statistic	Std. Error	Std. Test Statistic	Sig.	Adj. Sig.[a]
MC-Uncoated	22.867	7.422	3.081	0.002	0.021
MC-NC	-25.333	7.422	-3.413	0.001	0.006
MC-FC	30.100	7.422	4.056	0.000	0.000

MC-MCC	-41.033	7.422	-5.529	0.000	0.000
Uncoated-NC	-2.467	7.422	-0.332	0.740	1.000
Uncoated-FC	-7.233	7.422	-0.975	0.330	1.000
Uncoated-MCC	-18.167	7.422	-2.448	0.014	0.144
NC-FC	4.767	7.422	0.642	0.521	1.000
NC-MCC	15.700	7.422	2.115	0.034	0.344
FC-MCC	-10.933	7.422	-1.473	0.141	1.000

Each row tests the null hypothesis that the Sample 1 and Sample 2 distributions are the same.
Asymptotic significances (2-sided tests) are displayed. The significance level is ,050.
a. Significance values have been adjusted by the Bonferroni correction for multiple tests.

Day_0_Colour across Treatment_day

Pairwise Comparisons of Treatment_day

Sample 1-Sample 2	Test Statistic	Std. Error	Std. Test Statistic	Sig.	Adj. Sig.[a]
MC-NC	-2.400	6.967	-0.344	0.730	1.000
MC-Uncoated	7.267	6.967	1.043	0.297	1.000
MC-FC	9.700	6.967	1.392	0.164	1.000
MC-MCC	-12.133	6.967	-1.742	0.082	0.816
NC-Uncoated	4.867	6.967	0.699	0.485	1.000
NC-FC	7.300	6.967	1.048	0.295	1.000
NC-MCC	9.733	6.967	1.397	0.162	1.000

Uncoated-FC	-2.433	6.967	-0.349	0.727	1.000
Uncoated-MCC	-4.867	6.967	-0.699	0.485	1.000
FC-MCC	-2.433	6.967	-0.349	0.727	1.000

Each row tests the null hypothesis that the Sample 1 and Sample 2 distributions are the same.
Asymptotic significances (2-sided tests) are displayed. The significance level is ,050.
a. Significance values have been adjusted by the Bonferroni correction for multiple tests.

Pairwise Comparisons of Treatment_day

Sample 1-Sample 2	Test Statistic	Std. Error	Std. Test Statistic	Sig.	Adj. Sig.[a]
Uncoated-FC	-3.200	7.511	-0.426	0.670	1.000
Uncoated-MC	-11.600	7.511	-1.544	0.123	1.000
Uncoated-NC	-16.400	7.511	-2.183	0.029	0.290
Uncoated-MCC	-23.300	7.511	-3.102	0.002	0.019
FC-MC	-8.400	7.511	-1.118	0.263	1.000
FC-NC	-13.200	7.511	-1.757	0.079	0.789
FC-MCC	-20.100	7.511	-2.676	0.007	0.075
MC-NC	-4.800	7.511	-0.639	0.523	1.000
MC-MCC	-11.700	7.511	-1.558	0.119	1.000
NC-MCC	6.900	7.511	0.919	0.358	1.000

Each row tests the null hypothesis that the Sample 1 and Sample 2 distributions are the same.
Asymptotic significances (2-sided tests) are displayed. The significance level is ,050.
a. Significance values have been adjusted by the Bonferroni correction for multiple tests.

Day_6_Colour across Treatment_day

Pairwise Comparisons of Treatment_day

Sample 1-Sample 2	Test Statistic	Std. Error	Std. Test Statistic	Sig.	Adj. Sig.[a]
FC-Uncoated	3.500	7.631	0.459	0.646	1.000
FC-MC	-14.000	7.631	-1.835	0.067	0.666
FC-NC	-21.133	7.631	-2.769	0.006	0.056
FC-MCC	-23.367	7.631	-3.062	0.002	0.022
Uncoated-MC	-10.500	7.631	-1.376	0.169	1.000
Uncoated-NC	-17.633	7.631	-2.311	0.021	0.208
Uncoated-MCC	-19.867	7.631	-2.603	0.009	0.092
MC-NC	-7.133	7.631	-0.935	0.350	1.000
MC-MCC	-9.367	7.631	-1.227	0.220	1.000
NC-MCC	2.233	7.631	0.293	0.770	1.000

Each row tests the null hypothesis that the Sample 1 and Sample 2 distributions are the same.
Asymptotic significances (2-sided tests) are displayed. The significance level is ,050.
a. Significance values have been adjusted by the Bonferroni correction for multiple tests.
